# COVID-19 Mitigation Behaviors by Age Group — United States, April–June 2020

**DOI:** 10.15585/mmwr.mm6943e4

**Published:** 2020-10-30

**Authors:** Helena J. Hutchins, Brent Wolff, Rebecca Leeb, Jean Y. Ko, Erika Odom, Joe Willey, Allison Friedman, Rebecca H. Bitsko

**Affiliations:** ^1^CDC COVID-19 Response Team; ^2^Data Foundation, Washington, DC; ^3^Division of Human Development and Disability, National Center on Birth Defects and Developmental Disabilities, CDC.

*On October 27, 2020, this report was posted online as an *MMWR *Early Release.*

CDC recommends a number of mitigation behaviors to prevent the spread of SARS-CoV-2, the virus that causes coronavirus disease 2019 (COVID-19). Those behaviors include 1) covering the nose and mouth with a mask to protect others from possible infection when in public settings and when around persons who live outside of one’s household or around ill household members; 2) maintaining at least 6 feet (2 meters) of distance from persons who live outside one’s household, and keeping oneself distant from persons who are ill; and 3) washing hands often with soap and water for at least 20 seconds, or, if soap and water are not available, using hand sanitizer containing at least 60% alcohol (*1*). Age has been positively associated with mask use (*2*), although less is known about other recommended mitigation behaviors. Monitoring mitigation behaviors over the course of the pandemic can inform targeted communication and behavior modification strategies to slow the spread of COVID-19. The Data Foundation COVID Impact Survey collected nationally representative data on reported mitigation behaviors during April–June 2020 among adults in the United States aged ≥18 years (*3*). Reported use of face masks increased from 78% in April, to 83% in May, and reached 89% in June; however, other reported mitigation behaviors (e.g., hand washing, social distancing, and avoiding public or crowded places) declined marginally or remained unchanged. At each time point, the prevalence of reported mitigation behaviors was lowest among younger adults (aged 18–29 years) and highest among older adults (aged ≥60 years). Lower engagement in mitigation behaviors among younger adults might be one reason for the increased incidence of confirmed COVID-19 cases in this group, which have been shown to precede increases among those >60 years (*4*). These findings underscore the need to prioritize clear, targeted messaging and behavior modification interventions, especially for young adults, to encourage uptake and support maintenance of recommended mitigation behaviors to prevent the spread of COVID-19.

The COVID Impact Survey collected data to provide national estimates of health, economic, and social well-being of U.S. adults, using a national probability sample covering approximately 97% of the U.S. population of non-institutionalized adults with a home address (*3*). Surveys were conducted in three waves (April 20–26, May 4–10, and May 30–June 8), without significant resampling of persons across waves. Analyses included a total of 6,475 online or telephone surveys of adults aged ≥18 years.* The response rate among those invited to participate ranged from 19.7% to 26.1% across the three survey waves. Following data collection, an iterative raking process was used to adjust for nonresponse, noncoverage, and under- and oversampling (*5*). Demographic weighting variables provided in the dataset were obtained from the 2020 Current Population Survey; estimates reflect the U.S. household population of adults aged ≥18 years.^†^ No personally identifying information was provided in the data file accessed by CDC.^§^

Respondents were asked, “Which of the following measures, if any, are you taking in response to the coronavirus?*”* Of the 19 response options, three mitigation behaviors aligning with CDC recommendations were assessed: 1) “wore a face mask,” 2) “washed or sanitized hands,” and 3) “kept six feet distance from those outside my household.”^¶^ Three social mitigation behaviors aligning with CDC considerations and White House guidelines from March and April 2020 also were selected for analysis: 1) “avoided public or crowded places,” 2) “cancelled or postponed social or recreational activities,” and 3) “avoided some or all restaurants.”**^,††,§§^ Pearson's Chi-squared test was used to assess differences in reported behaviors (individual and cumulative) by age, within each survey wave and stratified by face mask use, based on a significance level of α = 0.05. Logistic regression models were used to test statistical significance of time trends by assigning calendar week of data collection for each survey wave as a single linear predictor for individual and cumulative behavioral outcomes. All analyses were conducted in Stata ES (version 16.1, StataCorp.) with survey weights applied during analyses for nationally representative estimates.

Across survey waves, the majority of the weighted sample (range = 62%–65%) identified as Non-Hispanic or Latino White, and 50% identified as female; 14%–15% of respondents were aged 18–29 years. In April, 78% of adults aged ≥18 years reported wearing a mask; this increased to 83% in May and 89% in June (Table 1) (p<0.001). All other reported mitigation behaviors decreased from April 20–26 to early June (p<0.05), except avoiding some or all restaurants, which did not change significantly (Table 1) (Supplementary Figure 1: https://stacks.cdc.gov/view/cdc/95944). At each time point, >40% of all adults aged ≥18 years reported all six assessed mitigation behaviors (Table 2). Across all survey waves, reported prevalences of mitigation behaviors were highest among adults aged ≥60 years and lowest among those aged 18–29 years (Table 1) (Supplementary Figure 1: https://stacks.cdc.gov/view/cdc/95944). Age was also significantly associated with the cumulative number of reported mitigation behaviors across all survey waves, with young adults reporting engaging in fewer mitigation behaviors compared with older adults overall and at all time points (Table 2) (Figure).

**TABLE 1:** Self-reported mitigation behaviors,* by adult age group — COVID Impact Survey, United States, April–June 2020^†^

Behavior/Characteristic	Wave 1 Apr (N = 2,190)			Wave 2 May (N = 2,238)			Wave 3 Jun (N = 2,047)	
	Yes (No.)	Weighted % (95% CI)		Yes (No.)		Weighted % (95% CI)	Yes (No.)	Weighted % (95% CI)
**Wore a face mask** ^§^								
**Total**	1,713	78.1 (76.1–80.1)		1,855		82.9 (81.3–84.4)	1,815	88.7 (87.2–90.0)
**Age group (yrs)**								
18–29	195	69.6 (63.3–75.3)^†^		261		81.8 (77.2–85.7)	273	86.1 (81.9–89.5)^†^
30–44	506	74.7 (70.7–78.4)		542		83.1 (80.1–85.8)	538	86.4 (83.4–88.8)
45–59	419	79.8 (75.6–83.65)		431		80.7 (77.1–83.8)	406	88.3 (85.0–90.9)
≥60	593	83.7 (80.3–86.6)		621		84.7 (81.9–87.2)	598	92.4 (90.1–94.2)
**Washed or sanitized hands** ^§^								
**Total**	2,037	93.1 (91.8–94.2)		2,043		91.3 (90.1–92.4)	1,828	89.3 (87.9–90.6)
**Age (yrs)**								
18–29	236	83.5 (77.9–87.8)^†^		281		88.1 (84.1–91.2)^†^	259	81.7 (77.1–85.6)^†^
30–44	615	91.6 (88.9–93.7)		577		88.5 (85.8–90.7)	540	86.7 (83.8–89.1)
45–59	499	95.0 (92.4–96.8)		497		93.1 (90.6–94.9)	429	93.3 (90.6–95.2)
≥60	687	96.5 (94.6–97.7)		688		93.9 (91.9–95.4)	600	92.7 (90.5–94.5)
**Kept 6 feet distance** ^§^								
**Total**	1,913	87.4 (85.7–88.9)		1,924		86.0 (84.5–87.4)	1,683	82.2 (80.5–83.8)
**Age group (yrs)**								
18–29	202	71.7 (65.5–77.2)^†^		245		76.8 (71.9–81.1)^†^	225	71.0 (65.7–75.7)^†^
30–44	565	84.6 (81.2–87.5)		541		83.0 (79.9–85.7)	490	78.7 (75.3–81.7)
45–59	486	93.1 (90.3–95.1)		468		87.6 (84.6–90.2)	386	83.9 (80.3–87.0)
≥60	660	92.6 (90.0–94.5)		670		91.4 (89.2–93.2)	582	90.0 (87.4–92.0)
**Cancelled/postponed pleasure, social, or recreational activities** ^§^								
**Total**	1,554		69.8 (67.5, 71.9)		1,514	67.8 (65.9, 69.7)	1,291	63.1 (61.0, 65.1)
**Age group (yrs)**								
18–29	167		60.0 (53.4–66.3)^†^		208	65.2 (59.8–70.2)	185	58.4 (52.9–63.7)^†^
30–44	474		68.5 (64.2–72.4)		435	66.7 (63.0–70.2)	377	60.5 (56.6–64.3)
45–59	376		70.3 (65.5–74.6)		363	68.0 (63.9–71.8)	292	63.5 (59.0–67.8)
≥60	537		74.8 (70.9–78.3)		508	69.3 (65.9–72.5)	437	67.5 (63.8–71.0)
**Avoided public or crowded places** ^¶^								
**Total**	1,762		80.5 (78.5–82.4)		1,724	77.0 (75.2–78.7)	1,542	75.3 (73.4–77.2)
**Age group (yrs)**								
18–29	204		74.2 (68.0–79.5)^†^		238	74.6 (69.5–79.1)^†^	213	67.2 (61.8–72.1)^†^
30–44	511		75.5 (71.5–79.1)		494	75.8 (72.3–78.9)	454	72.9 (69.2–76.2)
45–59	432		82.8 (78.7–86.2)		400	74.9 (71.1–78.4)	346	75.2 (70.1–79.0)
≥60	615		86.3 (83.1–89.0)		592	80.8 (77.8–83.5)	529	81.8 (78.6–84.6)
**Avoided some or all restaurants**								
**Total**	1,574		71.9 (69.6–74.0)		1,578	70.5 (68.6–72.4)	1,446	70.6 (68.6–72.6)
**Age group (yrs)**								
18–29	113		60.4 (53.8–66.6)^†^		217	68.0 (62.7–72.9)	201	63.4 (58.0–68.5)^†^
30–44	465		68.2 (64,0–72.2)		470	72.1 (68.5–75.4)	419	67.3 (63.5–70.8)
45–59	148		73.7 (69.2–77.8)		357	66.9 (62.8–70.7)	327	71.1 (66.8–75.1)
≥60	148		78.9 (75.3–82.2)		534	72.9 (69.5–76.0)	499	77.1 (73.7–80.2)

**Abbreviations:** CI = confidence interval; COVID-19 = coronavirus disease 2019.

* Wore a face mask, washed or sanitized hands, kept 6 feet of distance, avoided public or crowded places, canceled or postponed pleasure, social, or recreational activities and avoided some or all restaurants.

^†^ Chi-square p-value <0.05 for differences across age groups, by survey wave.

^§^ Test for trend for overall change over time: p-value <0.001.

**^¶^** Test for trend for overall change over time for “avoided public or crowded places”: p-value = 0.002.

**TABLE 2:** Cumulative number of self-reported mitigation behaviors,* by adult age group^†^ — COVID Impact Survey, United States, April–June 2020

Characteristics	0–1 Mitigation behaviors		2–3 Mitigation behaviors		4–5 Mitigation behaviors		All 6 Mitigation behaviors	
	No.	Weighted % (95% CI)	No.	Weighted % (95% CI)	No.	Weighted % (95% CI)	No.	Weighted % (95% CI)
**Combined survey waves (N = 6,475)** ^§^								
**Total**	383	6.2 (5.6–6.8)	848	13.2 (12.4–14.1)	2,309	35.4 (34.2–36.6)	2,935	45.2 (44.0–46.5)
**Age group (yrs)**								
18–29	97	10.5 (8.6–12.8)	153	15.7 (13.4–18.4)	350	38.1 (34.8–41.4)	318	35.7 (32.5–39.0)
30–44	148	7.9 (6.7–9.3)	288	15.0 (13.3–16.7)	662	33.3 (31.1–35.5)	849	43.9 (41.6–46.3)
45–59	76	5.5 (4.4–6.9)	190	12.8 (11.1–14.7)	556	35.6 (33.1–38.2)	696	46.1 (43.4–48.7)
≥60	62	3.1 (2.4–4.0)	217	10.7 (9.4–12.2)	741	36.0 (33.8–38.2)	1,072	50.2 (48.0–52.5)
**Wave 1 Apr (N = 2,190)** ^§^								
**Total**	109	5.3 (4.3–6.5)	265	11.6 (10.2–13.2)	827	38.6 (36.2–40.9)	989	44.5 (42.1–46.9)
**Age group (yrs)**								
18–29	33	12.4 (8.6–17.6)	52	15.2 (11.2–20.2)	115	42.7 (36.4–49.3)	82	29.7 (24.1–36.1)
30–44	45	7.3 (5.3–10.0)	96	14.5 (11.7–17.9)	251	37.1 (33.0–41.5)	280	41.1 (36.8–45.5)
45–59	14	2.7 (1.5–4.7)	58	11.0 (8.3–14.5)	215	40.7 (36.0–45.7)	237	45.6 (40.7–50.5)
≥60	17	2.3 (1.4–4.0)	59	7.8 (5.8–10.3)	246	36.5 (32.5–40.7)	390	53.4 (49.2–57.6)
**Wave 2 May (N = 2,238)^¶^**								
**Total**	115	5.1 (4.3–6.1)	299	13.4 (12.0–14.8)	842	37.6 (35.6–39.7)	982	43.9 (41.8–45.9)
**Age (yrs)**								
18–29	28	8.8 (6.1–12.4)	42	13.2 (10.0–17.3)	132	41.4 (36.1–46.9)	117	36.7 (31.6–42.1)
30–44	41	6.3 (4.7–8.4)	90	13.8 (11.4–16.7)	233	35.7 (32.2–39.5)	288	44.2 (40.4–48.0)
45–59	27	5.1 (3.5–7.3)	76	14.2 (11.5–17.5)	195	36.5 (32.5–40.7)	236	44.2 (40.0–48.4)
≥60	19	2.6 (1.7–4.0)	91	12.4 (10.2–15.0)	282	38.5 (35.0–42.1)	341	46.5 (42.9–50.1)
**Wave 3 Jun (N = 2,047)** ^§^								
**Total**	159	7.8 (6.7–9.0)	284	13.9 (12.4–15.4)	640	31.3 (29.3–33.3)	964	47.1 (44.9–49.3)
**Age group (yrs)**								
18–29	36	11.4 (8.3–15.3)	59	18.6 (14.7–23.3)	103	32.5 (27.6–37.8)	119	37.5 (32.4–43.0)
30–44	62	10.0 (7.8–12.6)	102	16.4 (13.7–19.5)	178	28.6 (25.2–32.3)	281	45.1 (41.2–49.0)
45–59	35	7.6 (5.5–10.4)	56	12.2 (9.5–15.5)	146	31.7 (27.7–36.1)	223	48.5 (43.9–53.1)
≥60	26	4.0 (2.8–5.8)	67	10.4 (8.2–13.0)	213	32.9 (29.4–36.6)	341	52.7 (48.9–56.5)

**Abbreviations:** CI = confidence interval; COVID-19 = coronavirus disease 2019.

* Wore a face mask, washed or sanitized hands, kept 6 feet of distance, avoided public or crowded places, canceled or postponed pleasure, social, or recreational activities and avoided some or all restaurants.

^†^ Test for trend for overall change in behavior over time p-value <0.001.

^§^ Chi-square p-value <0.01 for differences across age groups, by survey wave.

**^¶^** Chi-squared p*-*value <0.05 for differences across age groups, by survey wave.

**FIGURE:**
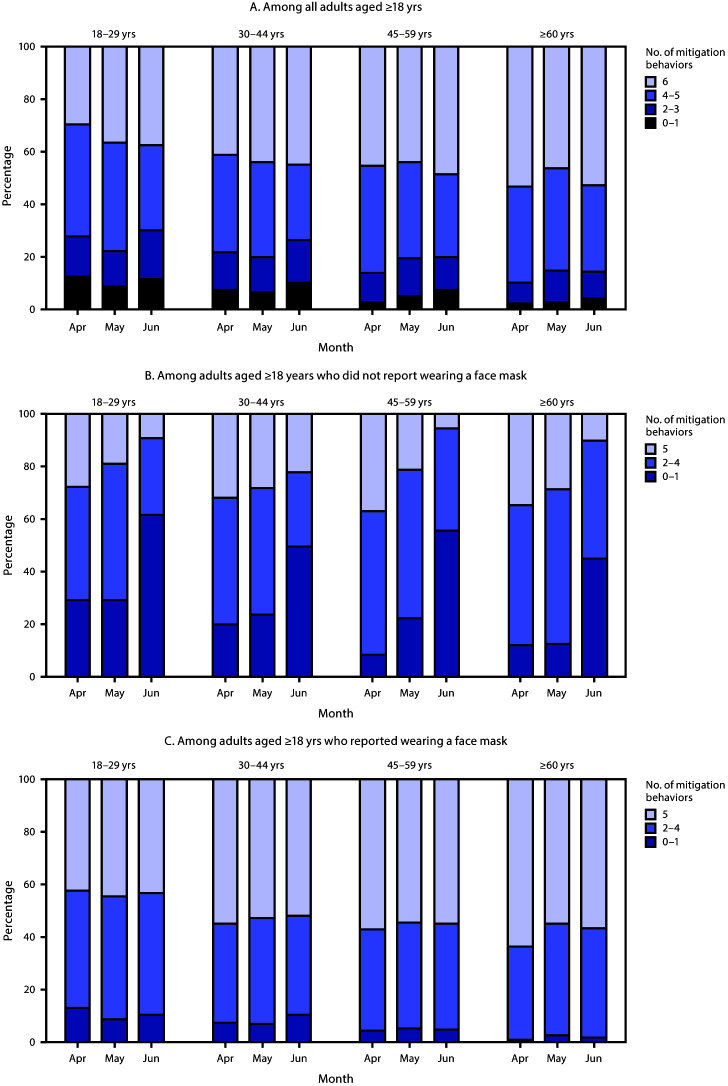
Percentage distribution of cumulative number of reported mitigation behaviors,* by age group and reported face mask use — COVID Impact Survey, United States, April–June 2020^†^^,^^§^^,^^¶^^,^^**^^,^^††^^,^^§§^ **Abbreviation:** COVID-19 = coronavirus disease 2019. * Wore a face mask; washed or sanitized hands; kept 6 feet of distance; avoided public or crowded places; canceled or postponed pleasure, social, or recreational activities; and avoided some or all restaurants. ^†^ Weighted to be representative of noninstitutionalized U.S. adults; values <5% not shown. ^§^ Trend for overall change in behavior over time, p-value <0.001. ^¶^ Chi-squared p-value <0.001 for differences in cumulative number of mitigation behaviors reported across age groups, within all survey waves. ** Chi-squared p-value <0.05 for differences in cumulative number of mitigation behaviors reported across age groups, within April and June waves only. ^††^ Not inclusive of the survey item “wore a face mask.” ^§§^ Trend for overall change in behavior over time p-value <0.05 (among those who reported wearing a mask: p-value = 0.003).

Among adults who reported face mask use at each time point, a significantly higher percentage reported other mitigation behaviors compared with those who did not report mask use (Supplementary Figure 2: https://stacks.cdc.gov/view/cdc/95945). Among adults who did not report mask use, all other reported mitigation behaviors declined significantly from the April 20–26 wave to early June. Other mitigation behaviors also decreased over time among those who reported mask use, but to a much lesser extent, and only significantly for washing hands, maintaining a 6-foot distance, and cancelling or postponing social events. A higher percentage of adults who reported mask use also reported a higher cumulative number of other mitigation behaviors during the same period, compared with adults who did not report mask use (Figure). By early June, >45% of adults who did not report mask use reported one or fewer other mitigation behaviors (Figure). Overall, a significant positive association between age and the cumulative number of reported mitigation behaviors persisted over time among those who did and those who did not report mask use (Figure).

## Discussion

This report provides four important insights into the practice of mitigation behaviors among U.S. adults to prevent the spread of SARS-CoV-2. First, the majority of U.S. adults reported engaging in most or all of the six mitigation behaviors assessed. Second, age was an important determinant of engagement in mitigation behaviors overall. A smaller percentage of adults aged <60 years, particularly those aged 18–29 years, reported engaging in the mitigation behaviors assessed compared with adults aged ≥60 years. Third, while reported use of face masks increased significantly across all age groups over time, other reported mitigation behaviors declined or did not change significantly across age groups. Finally, compared with adults who reported wearing a mask, those who did not report mask use also reported engaging in significantly fewer other mitigation behaviors during the same period, with significant declines in all other behaviors from April to June.

CDC recommends multiple, concurrent mitigation behaviors to most effectively reduce the spread of COVID-19 (*6*). Fewer reported mitigation behaviors among young adults might contribute to the high incidence of confirmed COVID-19 cases in this age group (*4*). Older adults might be more concerned about COVID-19, based on their higher risk for severe illness compared with that of younger adults (*7*). Young adults might also be less likely to engage in mitigation behaviors because of social, developmental, and practical factors (*8*,*9*). Across age groups, increases in mask use and decreases in other mitigation behaviors might reflect elevated promotion of mask use over time, along with the lifting of shelter-in-place orders and reopening of business, service, hospitality and other sectors.

Significant declines in self-reported mitigation behaviors among those not reporting mask use suggests that a minority of persons might be increasingly resistant to COVID-19 mitigation behaviors or unable to engage in mitigation behaviors because of the constraints introduced by their return to work, school, or other settings. Effectively promoting engagement in mitigation behaviors among young adults will require moving beyond education to addressing barriers to mitigation behaviors as the pandemic and the response evolve. Strategies might include engaging trusted leaders and social media influencers to improve social acceptability of mitigation behaviors, offering practical tips for engagement, and appealing to personal values. Strategies also might include addressing social and emotional challenges potentially associated with social distancing behaviors, and engaging communities, businesses, employers and institutes of higher education to ensure mitigation behaviors are both feasible and actively encouraged where young adults work, study, and engage in recreational activities. Similar targeted strategies can be used to promote use of recommended mitigation behaviors among all adults.

The findings in this report are subject to at least three limitations. First, survey questions did not ask about consistency, adequacy, or frequency of mitigation behaviors in alignment with public health recommendations and thus might overestimate the real prevalence of mitigation behaviors. For example, the survey item “wore a face mask” did not ask whether a mask was worn over the nose and mouth in public settings and when around persons who are ill or those who live outside of one’s household. Similarly, the survey item “washed or sanitized hands” did not specify frequency of handwashing or handwashing in situations associated with higher risk of exposure to SARS-CoV-2 (e.g., while in a public place) nor did it specify that hands were washed often with soap and water for at least 20 seconds or that sanitizer containing at least 60% alcohol was used. Second, the survey item “avoided some or all restaurants” did not specify type of restaurant service (e.g., curbside pick-up versus dining in), which might underestimate risk mitigation, as on-site dining has been associated with an increased risk for acquiring COVID-19 (*10*). Finally, all results depend on self-report and thus social desirability and recall bias might result in over- or underestimation of reported mitigation behaviors.

These findings suggest that lower engagement in social mitigation behaviors among younger adults might be one possible reason for the increased incidence of confirmed COVID-19 cases in this group, which began in June 2020 and preceded increases among persons aged ≥60 years by 4–15 days (*4*). Better understanding of barriers and motivators associated with participation in mitigation behaviors is needed to effectively employ strategies that promote engagement of younger adults and others who are not currently engaging in mitigation behaviors. Reaching these groups through targeted channels, trusted leaders, and influencers at national, state, and local levels has the potential to improve use and effectiveness of critical public health strategies to protect persons of all ages by preventing the spread of SARS-CoV-2.

**Summary**

**What is already known about this topic?**

Recommended mitigation behaviors to prevent the spread of COVID-19 include wearing masks, hand washing, social distancing, and staying home when ill.

**What is added by this report?**

Self-reported engagement in mitigation behaviors (mask wearing, handwashing, physical distancing, crowd and restaurant avoidance, and cancellation of social activities) differed significantly by adult age group. During April–June 2020, the prevalence of these behaviors was lowest among adults aged 18–29 years and highest among those aged >60 years. Whereas mask wearing increased over time, other reported mitigation behaviors decreased or remained unchanged.

**What are the implications for public health practice?**

Improved communication and policy priorities are needed to promote recommended COVID-19 mitigation behaviors, particularly among young adults.

## Footnotes

* The number of interviews conducted was 2,190 in April, 2,238 in May, and 2,047 in June 2020.

†
https://www.census.gov/programs-surveys/cps.html

§ The COVID-19 Impact Survey is conducted by NORC at the University of Chicago. The NORC Institutional Review Board (FWA00000142) reviewed and approved the study protocol to protect the rights and welfare of human subjects.

¶ Respondents could select all behaviors that applied. All 19 possible responses included 1) canceled a doctor appointment; 2) wore a face mask; 3) visited a doctor or hospital; 4) canceled or postponed work activities; 5) canceled or postponed school activities; 6) canceled or postponed dentist or other appointment; 7) canceled outside housekeepers or caregivers; 8) avoided some or all restaurants; 9) worked from home; 10) studied at home; 11) canceled or postponed pleasure, social, or recreational activities; 12) stockpiled food or water; 13) avoided public or crowded places; 14) prayed; 15) avoided contact with high-risk persons; 16) washed or sanitized hands; 17) kept six feet distance from those outside my household; 18) stayed home because I felt unwell; and 19) wiped packages entering my home.

** https://www.cdc.gov/coronavirus/2019-ncov/prevent-getting-sick/how-covid-spreads.html.

†† https://www.cdc.gov/coronavirus/2019-ncov/daily-life-coping/personal-social-activities.html.

§§ https://www.whitehouse.gov/wp-content/uploads/2020/03/03.16.20_coronavirus-guidance_8.5x11_315PM.pdf.
